# Compulsory Health Insurance Bill (Mill’s)

**Published:** 1917-05

**Authors:** 


### Compulsory Health Insurance Bill (Mill's).

Address made by Dr. JOHN D. BONNAR, of Buffalo, representing the Medical Society of Erie County, before Judiciary Committee of the State Senate, at Albany, March 7, 1917.

I have the honor and responsibility of appearing before you today at the request of the President of the Medical Society of Erie County, Dr. Irving W. Potter, in opposition to the measure now before you. This society, of some 700 members, at a recent meeting, called for consideration of this bill, most vigorously and, without a dissenting voice, passed a resolution condemning the measure as pernicious, impractical and wholly out of harmony with the ethics, wisdom and progress of the times.

Gentlemen, the origin of this bill being shrouded in mystery, as far as the public, at least the medical part thereof, is concerned—no known parents, or, if it has such, apparently they are of the illegitimate type, unwilling to acknowledge their offspring. That a senator has been found willing to act as foster-parent and introduce the measure, is surely because he failed to realize its inception. When but casually studied its nature is mysterious and full of terms, commissions, panels, councils, courts of higher and lower degree, arbitrators, and all the trumpery imaginable. It is out of harmony with order and the eternal fitness of things. It is plainly unsuited for any place upon the earth, much less for the enlightened citizenship of this great Empire State of New York. To even think of it is to cause such feeling of indignation, words fail, and our emotions silently spurn its vicious purpose of adorning the bill with a halo of "charity," while its true nature is treachery to human rights. Shall people of this twentieth century suffer such mockery in the name of progress, while its nature is treason to liberty? Never! This suggests collusion that gives us ample cause for full and plain analysis. If the measure were clearly in the welfare of any considerable proportion of our citizens, we should ask that its sponsors give reasons for this attempt to press into law what is, upon its face, so unusual and pernicious a measure—facts most palpable, even on casual study.

Estimating 10,000,000 as the population of this state, and some 25,000 medical practitioners, it is readily seen that the average number of clients for each practitioner in the state, would be 400—at most 500. Whereas, under this "compulsory insurance bill," the so-called "panel" physicians, conservatively estimated at 2-5 of the whole—would number ten thousand upon the ''panels.'' Some eighty per cent of the entire population, or 7,343,000, those receiving $100 or less per month wages, it is readily seen that each ''panel'' physician would have 800 patrons, while the other 3-5 of the doctors not accepting the lodge service, would have a clientele of 2,167,000, or on the average to each doctor of 133 persons. In the last analysis, however, it is seen that just about 1-5, rather than 2-5, nominally to be on the ''panels'' would have the entire burden of this whole ''panel'' or lodge (''work contract'') practice, in its worst form, which would thus increase the clientele from 800 to 1,600, for each so-called ''panel'' physician. As 10 days' sickness per person per annum is the average, as figured by actuaries, we see that 1,600 patrons at ten days each, needing a visit per day if properly treated, would mean just 16,000 visits to be made by the ''panel'' doctor per annum. Coupled with this, also some 15 office calls per day, from such sick people able to come to the office, there would be added about 5,000 office visits required by the ''panel'' doctors per annum. This is wholly impossible as to stamp the measure as visionary and absurd. It would, however, at an estimated salary of $3,000 for such lodge doctor, give him per visit the munificent sum of 16 to 18 cents per visit, or 26 cents if he got $5,000 per annum.

Les than the aforementioned number of visits would be a neglect of the patient, while the making of such by the physician would amount to slavery, hence either the patient or the ''panel'' physician would be the victim of plain injustice.

Such a measure as this strikes at the very foundation of our constitutional rights of life, liberty and the pursuit of happiness. It is an open and palpable attempt to place upon the shoulders of the medical profession the onerous duty of giving of their time, skill and intelligence, for a mere pittance, the needed medical, surgical and obstetric care of four-fifths of the population of the state. Such a measure has no claim in equity nor justice, nor even in any scheme whatever of social economy. It is an opprobrium—a veritable marplot against liberty. It attempts by its commissions, boards of arbitration, and a dozen other new and wholly foreign functions, to hide its real mischief-throttling of freedom—an attempt to enslave a portion of the medical profession, in a most ingenious manner, by putting them in the light of ''contestants'' for what,under our principles of citizenship, is their inalienable right. Conceived, as it plainly is, in sin and (if it ever sees the light of day), brought forth in iniquity, we trust, gentlemen of this judiciary committee of the Senate of the great state of New York, that you will see to it that this measure never comes out of your committee, as it is arbitrary, autocratic and entirely devoid of the democratic spirit, upon which this government is founded. On the other hand, if it ever sees the light of day, it will prove a monstrosity upon the body politic, and cast a dark cloud upon its fair escutcheon.

Note—This measure was voted down in the Judiciary Committee of the Senate, but, of course, may appear in a new garb at some later time, therefore needs watching—J. D. B.

---

**Amoebic Dysentery.** H. W. Soper of St. Louis, Int. Clin., Vol. 4, Series 26, in a general article on the use of the sigmoidoscope, examined 30 cases during treatment by ipecac and emetine. All cases showed the typic lesions in the rectum or sigmoid and, in some, the diagnosis was made when the amoebae could not be demonstrated. The lesions may persist after clinical cure. Insufflations of 1:10 tannin and bismuth, heal the lesions quickly. Ipecac powder, even much diluted, caused severe inflammation without destroying the amoebae. (Note: The simplicity and rapidity of relief of various local applications through the sigmoidoscope is surprising. Even occult blood may come from an ulcer only 10 inches above the anus. Major conditions are usually diagnosticable without endoscopy and, unless inoperable, cannot be adequately treated through the speculum. Thus the diagnostic and therapeutic use of the sigmoidoscope is reduced mainly to very simple conditions, scarcely worth reporting from one point of view, yet when one considers the relief afforded and the obstinacy of these cases of almost insignificant inflammation and ulceration, more attention ought to be paid to this means. The writer has used the lateral posture and has discarded the window and most of the pneumatic devices, although occasionally air is pumped ahead of the instrument through a metal tube fixed in the obturator. Reflected light is inferior to direct, introduced by a slender, movable and removable staff. Discarding the complications of posture, pneumatics and reflected light, the sigmoidoscope is a very simple instrument. One cannot even reduce the manipulations to a system, on account of differences in the anatomy of the valves, etc. But, even with patience, one cannot always introduce it if he has respect for the danger of forcible manipulation.—Ed.)

---

**Roentgen Treatment of Exopthalmic Goitre and Hyperthyroidism.** C. A. Simpson, Wash. Med. Annals, March, reports 17 cases, all successful but 4, of which one was cancerous.

